# Immunocytometric analysis of patients with thymic epithelial tumors revealed that COVID-19 vaccine booster strongly enhanced the immune response

**DOI:** 10.3389/fimmu.2023.1233056

**Published:** 2023-08-29

**Authors:** Gustavo Cernera, Monica Gelzo, Pietro De Placido, Margaret Ottaviano, Erica Pietroluongo, Maddalena Raia, Giulia Scalia, Marianna Tortora, Giuseppe Castaldo, Pietro Formisano, Giovannella Palmieri, Mario Giuliano

**Affiliations:** ^1^ CEINGE-Biotecnologie avanzate, scarl, Naples, Italy; ^2^ Dipartimento di Medicina Molecolare e Biotecnologie Mediche, Università di Napoli Federico II, Naples, Italy; ^3^ Dipartimento di Medicina Clinica e Chirurgia, Università di Napoli Federico II, Naples, Italy; ^4^ Dipartimento di Melanoma, Immunoterapia Oncologica e Terapie Innovative, IRCCS Fondazione G. Pascale, Naples, Italy; ^5^ Centro Regionale di Coordinamento Tumori Rari Regione Campania (CRCTR), Naples, Italy; ^6^ Dipartimento di Scienze Mediche Traslazionali, Università di Napoli Federico II, Naples, Italy

**Keywords:** COVID-19, vaccine, booster, thymic epithelial tumors (TETs), immunophenotype, humoral response, cell-mediated response

## Abstract

**Background:**

Thymic epithelial tumors (TETs) are rare malignancies with heterogeneous clinical manifestations. The high frequency of autoimmune paraneoplastic disorders observed in such patients requires caution when using COVID-19 vaccines. Furthermore, TETs are often associated with severe immunodeficiency, making it difficult to predict vaccine immunization. Therefore, we aimed to evaluate immune response to COVID-19 vaccine in patients with TETs.

**Methods:**

We conducted a prospective study enrolling patients who underwent the SARS-Cov-2 mRNA full vaccine cycle (two doses plus a booster after 6 months of BNT162b2). All patients were enrolled before receiving 1^st^ vaccine dose and were followed over the vaccination cycle for up to 6 months after the booster dose to i) assess humoral and cellular responses, ii) define biomarkers predictive of effective immunization, and iii) evaluate the safety of the vaccine.

**Results:**

At the end of the full vaccine cycle, 27 (61.4%) patients developed humoral and 38 (86.4%) cellular responses (IFN γ release by stimulated cells) and showed an increase in activated TH1 and TH17 cells, particularly significant after the booster dose. The number of B and T lymphocytes at baseline was predictive of humoral and cellular responses, respectively. Patients with no evidence of tumor lesions had a higher probability of achieving a humoral response than those with evidence of the disease. Furthermore, the percentage of patients with immune-related disorders (75%), particularly Good’s syndrome (47.7%) and myasthenia gravis (29.5%), did not change over the entire vaccine cycle. Overall, 19 of the 44 enrolled patients (43.2%) had COVID-19 during the observation period; none required hospitalization or oxygen support, and no fatalities were observed.

**Conclusion:**

SARS-Cov-2 mRNA vaccine determines the immune responses in patients with TET, particularly after the booster dose, and in patients with no evidence of tumor lesions. Preliminary analysis of B and T lymphocytes may help identify patients who have a lower probability of achieving effective humoral and cellular responses and thus may need passive immunization. The vaccine prevented severe COVID-19 infection and is safe.

## Introduction

In March 2020, the severe acute respiratory syndrome coronavirus 2 (SARS-CoV-2) became a pandemic that caused approximately seven million deaths. A tremendous effort of researchers permitted to obtain antiviral vaccines (1) since December 2020. However, mutations in the viral genome cause the onset of variants of interest (VOI) which quickly spread worldwide (2). These variants succeeded each other, but vaccines designed against the spike protein of the *wilde-type* virus, although not protecting against reinfection, offered a protective role against the severe form of the disease (3). In Italy, risk groups among frail patients underwent a three-dose scheme that included a first dose followed by a second administration after 21 days, and a third booster dose after 6 months.

Among frail subjects are patients with thymic epithelial tumors (TETs), a very rare malignancy (0.23-0.30 cases/100,000 per year) that represents the most frequent neoplasia of the anterior mediastinum. TETs include a spectrum of forms, including thymoma, thymic carcinoma, and thymic neuroendocrine tumors (4). Paraneoplastic immune-related disorders due to impaired immunologic self-tolerance and the presence of autoreactive T cell render such patients more prone to immune stimulation (5), placing caution on the use of COVID-19 vaccines, as well as the known cross-reactions of antibodies against the SARS-CoV-2 spike protein with tissue proteins (6). Furthermore, TETs are frequently associated with severe immunodeficiency, mainly involving B cells and Ig production; however, a specific signature indicates the presence of more complex immunological abnormalities (7). Therefore, it is difficult to predict whether COVID-19 vaccines warrant protection against the virus in such complex patients, considering the lack of effective immunization biomarkers.

In fact, after vaccine introduction, serum antibody kinetics was used as a marker of immunization (8); however, it became clear that the antibody level was not an effective indicator of immunization (9, 10). SARS-CoV-2 specific memory T cells, particularly CD4^+^ cells, are considered the main effectors of long-term immune protection (11, 12). To date, different procedures have been used to evaluate the T cell response to vaccines, including the release of interferon (IFN) γ, tumor necrosis factor (TNF), α and interleukin (IL)-2 by T cell after stimulation with the spike protein from vaccinated subjects (13, 14). Most studies have concluded that there is a lack of correlation between humoral and cellular responses (15, 16).

In the present study, we prospectively enrolled patients with TET who underwent the SARS-Cov-2 mRNA vaccine and were followed during the full vaccine cycle to: i) evaluate the effects of the vaccine in terms of clinical complications and immunization, ii) assess the humoral and cellular responses to the vaccine, and iii) define biomarkers predictive of an effective immunization.

## Methods

### Study design and participants

The study was conducted according to the principles of the Declaration of Helsinki and was approved by the Ethical Committee of the University of Naples Federico II (approval number 76.21). All enrolled patients signed an informed consent. Consecutive patients with TET, who were referred to the Regional Coordination Center for Rare Tumors of Campania Region (CRCTR) at University Hospital Federico II in Naples, Italy, were prospectively enrolled between April 2021 and November 2021. All patients were enrolled before receiving the first dose of SARS-CoV-2 mRNA vaccine (BNT162b2, Pfizer-BioNTech). Study inclusion criteria comprised age greater or equal to 18 years, histological diagnosis of TET, known status (presence vs absence) of paraneoplastic immune-related disorders, known disease status, defined as evidence of disease (ED) or no residual tumor lesion/s [no evidence of disease (NED)]. Immune-related disorders were diagnosed before study enrolment using national recommendations (17). The most common immune-related disorders, including Good’s syndrome (GS) and Myasthenia Gravis (MG), were diagnosed using the following criteria:

GS diagnosis was defined by the presence of recurrent infections due to encapsulated bacteria, fungi, and viruses, hypogammaglobulinemia, low or absent B cells, abnormal CD4/CD8 T cell ratio, CD4 T cell lymphopenia and impaired T cell mitogenic responses (18); MG diagnosis was defined by the presence of clinical signs of ptosis, diplopia or muscle weakness, accompanied by a positive antibody directed against post-synaptic antigens, muscle cholinergic receptor (AChR), muscle tyrosine-kinase (MuSK) or low-density protein type 4 (LRP4) (19).

In order to assess humoral and cellular immune response following vaccine administration, a longitudinal analysis of SARS-CoV-2 spike-binding IgG antibody serum levels and immune phenotypes was performed at different time points: T0 (before the first vaccine dose), T1 (1 week after the second dose), T2 (1 month after the second dose), T3 (3 months +/- 2 weeks after second dose), T4 (before booster vaccine dose), and T5 (after booster vaccine dose). In addition, cell-mediated immune responses were assessed at T4 and T5. Various viral VOI were circulating during this period in Southern Italy (20). Clinical and anamnestic evaluations were performed every four weeks during and after the administration of the full vaccination cycle for up to 12 months from 1^st^ dose, in order to identify humoral and/or clinical signs or symptoms suggestive of new-onset or worsening autoimmune disorders. Furthermore, to assess the incidence and severity of COVID-19 infection in the patient cohort, we prospectively collected data on infection occurrence, need for oxygen support and/or hospitalization, and the overall clinical gravity of COVID-19 according to the National Institute of Health (NIH) classification (21) from the time of study enrollment to 6 months after the booster dose.

### Sample collection and storage

Blood samples were collected in EDTA tubes and processed within 3 h for antigen stimulation and cytokine release assays. Peripheral blood mononuclear cells (PBMCs) were collected by density gradient on lymphocyte separation media (Biowest) using Lymphosep tubes (Grenier Bio-One) according to the manufacturer’s instructions. Peripheral PBMCs were cryopreserved as previously described (20). Blood samples were collected in EDTA tubes for lymphocyte subpopulation analysis. Sera were obtained from blood samples in tubes with separation gels by centrifugation at 3500 rpm for 15 min and stored at -80°C until analysis.

### Antigen stimulation and cytokines release assay

To obtain the specific T cell response by the cytokine release assay, PBMCs were plated in a 96-U well plate at 1x10^6^ cells per well (100 μL) in complete RPMI medium with 5% human AB serum (Sigma) in the presence of 1 μg/mL monoclonal antibodies against CD28 and CD49d (Becton Dickinson) and stimulated as previously described with slight modifications (20). The SARS-CoV-2 protein S (Miltenyi Biotec) peptide pools were used at 1 ug. The peptide pool consists of 15-mer sequences with 11 amino acids overlap, covering the whole protein of spike glycoprotein (“S”) of SARS-Coronaviruses 2 Wuhan strain. Finally, IFN γ, IL-2 and TNF α levels were analyzed in the supernatants from PBMC. The assay was performed using the ELLA platform (Protein Simple). The stimulation Index (SI) was calculated as the ratio of the cytokine concentration produced after stimulation by the peptide pools to that of the control condition in each subject.

### Anti-SARS-CoV-2 IgG antibodies

Serum samples were analyzed for anti-SARS-CoV-2 IgG using the Liaison^®^ SARS-CoV-2 TrimericS IgG CLIA kit (DiaSorin^®^) according to the manufacturer’s protocol (22). Anti-SARS-CoV-2 IgG antibody levels are expressed in arbitrary units (AU/mL).

### Cytometric analysis

We used FACS Canto II (Becton Dickinson) with the previously described gating strategy (23–25) to evaluate lymphocyte subpopulations (23–25). Cell analysis was performed using Facs Diva software.

### Statistical analysis

Continuous data were reported as median and interquartile range (IQR). The normality of the distributions was evaluated using the Shapiro-Wilk test. Paired comparisons between two variables were performed using the Wilcoxon signed-rank test, whereas Friedman’s test was used for paired comparisons among the five times. Correlations between the variables were evaluated using Spearman’s correlation analysis. Partial least squares discriminant analysis (PLS-DA) was used to detect sample clustering in a supervised manner. Before PLS-DA, the data were normalized to the median of the raw values, log-transformed, and auto-scaled. The efficacy of the variables to discriminate non-responders from responders was evaluated using the area under the curve (AUC) obtained from the receiver operating characteristic (ROC) curve analysis (26). Statistical analyses were performed using SPSS (version 28, IBM SPSS Statistics) and MetaboAnalyst 5.0 online package [https://www.metaboanalyst.ca]. Graphics were generated using KaleidaGraph software (version 4.5.4, Synergy, Reading, PA, USA) and Graph Pad Prism 8 Software (GraphPad Software, San Diego, CA, USA). Statistical significance was set at P values < 0.01 were considered as significant.

## Results

### Patient clinical features evaluated before SARS-Cov-2 vaccine

Forty-four consecutive patients with TET were enrolled in the study. Patient and tumor characteristics at enrollment are shown in **Table 1**. Thirty-three (75.0%) patients had thymoma and 11 (25.0%) had thymic carcinoma. All patients had an Eastern Cooperative Oncology Group (ECOG) Performance Status (PS) ranging from 0 (n= 38; 86.4%) to 1 (n= 6; 13.6%). Overall, 33 patients (75%) had immune-related paraneoplastic disorders, with the most frequent being GS (n=21; 47.7%) and MG (n=13; 29.5%), while 6 patients (13.6%) had both MG and GS (**Table 2**). At the time of study enrollment, 24 patients (54.5%) were followed up with no residual tumor lesions and were referred to have no evidence of disease (NED), while the remaining 20 patients (45.5%) had evidence of disease (ED) and were receiving systemic treatment. Among the ED patients, 2 (10%) received platinum-based chemotherapy, 8 (40%) received etoposide-based chemotherapy, and 10 (50%) received octreotide LAR plus dexamethasone.

**Table 1:** Patient and tumor characteristics.

Characteristics	Number of patients (%)
Age, median (range)	55 (31 – 74)
Male	18 (41%)
Female	26 (59%)
ECOG PS	
0	38 (86.4%)
1	6 (13.6%)
Histological Type	
Thymoma A AB B1 B2 B1-B2 B2-B3 B3 Not otherwise specified Not available Thymic carcinoma	33 (75%) 2 (4.5%) 9 (2%) 3 (6.8%) 8 (18.2%) 1 (2.3%) 3 (2.3%) 5 (11.4%) 1 (2.3%) 1 (2.3%) 11 (25%)
Radiological Stage of disease according to TNM	
I II III IVA IVB	6 (13.6%) 7 (15.9%) 4 (9.1%) 12 (27.3%) 15 (34.1%)

ECOG PS, Eastern Cooperative Oncology Group Performance Status.

TNM: tumor, node, metastasis.

**Table 2:** Immune-related disorders.

Immune-related disorder	Number of patients (%)
Any immune-related disorder	33 (75%)
Good’s Syndrome (GS)	21 (47.7%)
Myasthenia Gravis (MG)	13 (29.5%)
Concomitant GS and MG	6 (13.6%)
Other immune-related disorders	5 (11.4%)

### Evaluation of humoral response by anti-SARS-CoV-2 IgG antibodies

**Figure 1:**
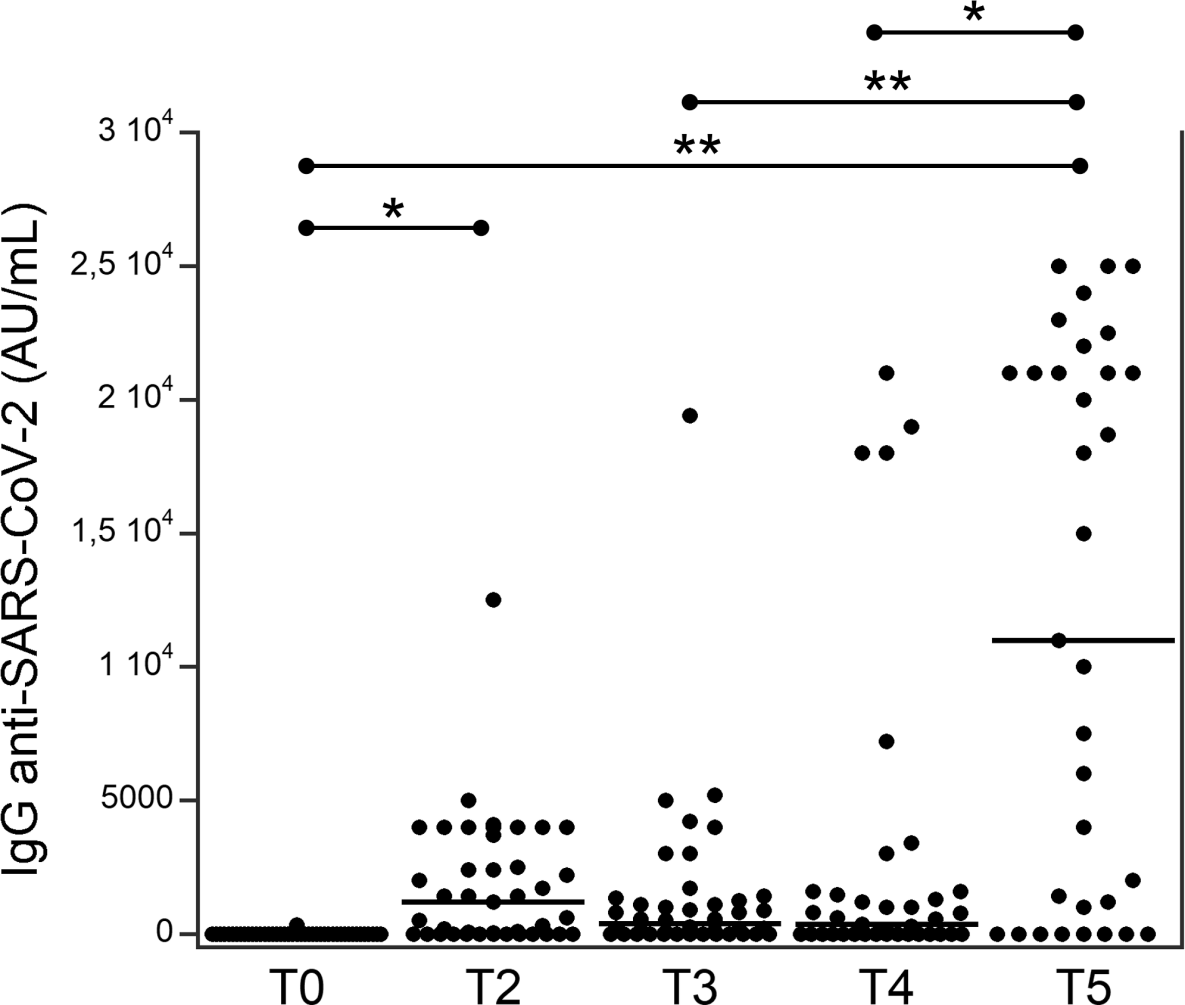
Serum levels of IgG (AU/ml) targeting the RBD in patients with thymic epithelial tumors at different times (see **Table 1**). *p<0.01; **p<0.001.

Among the 44 patients, 27 (61.4%) developed an IgG response and 17 (38.6%) did not develop a response after the second dose; the same 17 patients did not develop a response after the booster dose. **Figure 1** shows the serum levels of IgG (AU/mL) targeting RBD in patients with TETs at different times. At T2, the levels of serum IgG levels were significantly higher than they were at T0. Subsequently, they decreased at T3 and T4 and increased substantially at T5 as compared to at T0, T3, and T4.

### Evaluation of cellular response by antigen stimulation and cytokines release assay

**Figure 2:**
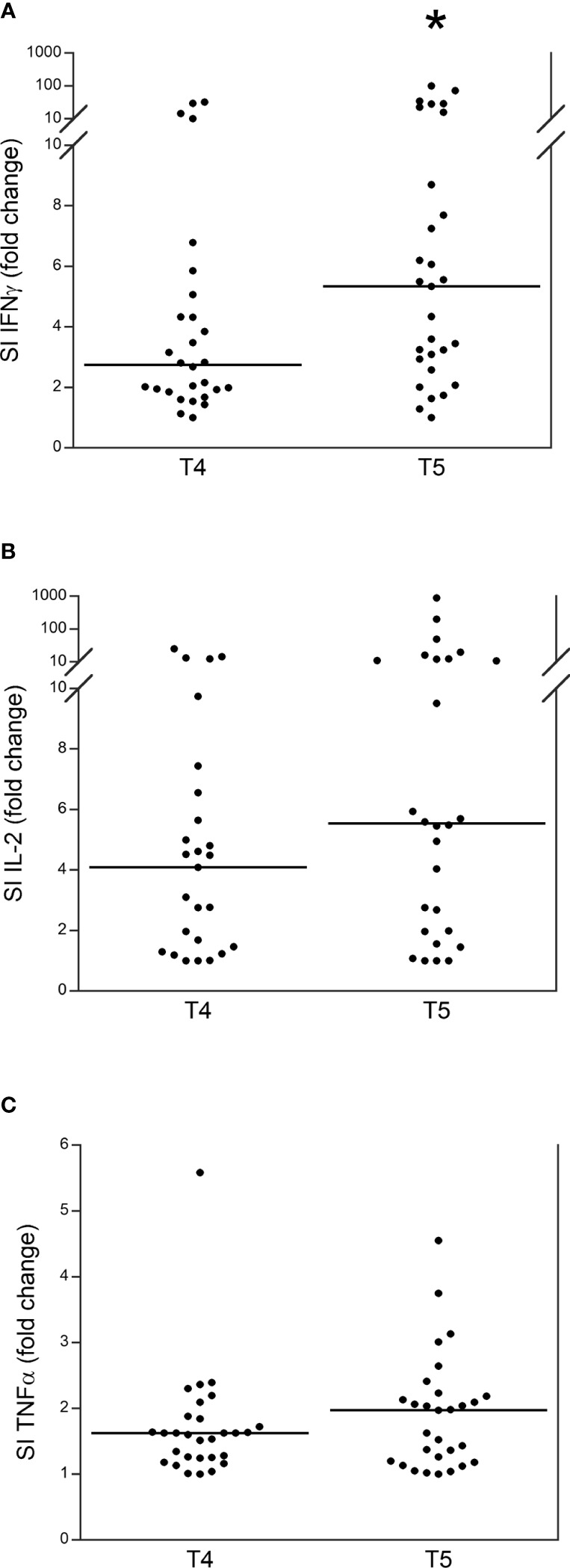
Release (stimulation index, SI) of IFN γ **(A)**, IL-2 **(B)** and TNF α **(C)** in patients with thymic epithelial tumors at T4 and T5. *p<0.01.

We assessed the release (SI) of IFN γ, IL-2, and TNF α by stimulating PBMCs from patients with TET at either T4 or T5. Among the 44 patients, 37 (84.1%) developed an IFN γ response after the booster dose, and seven (15.9%) did not develop a response, specifically four of these seven patients did not develop a humoral response, while three did. In particular, IFN γ release significantly increased at T5 than at T4 (**Figure 2A**), while IL-2 and TNF α release were higher in T5 than they were at T4, although not significantly (**Figures 2B, C**).

### White blood cell analysis by flow-cytometry

**Supplemental Table 1** shows the numbers of total blood leukocytes, neutrophils, lymphocytes, and platelets in patients with TET at T0, T2, T3, T4, and T5. None of these parameters changed significantly over time.

**Table 3** shows the lymphocyte populations in patients with TET at T0 compared to those obtained at T2, T3, T4, and T5. The number of B, NK, T, helper, and cytotoxic lymphocytes did not change significantly.

**Table 3:** Lymphocytes (N/mmc) in patients with TET from baseline to post-third dose of COVID-19 vaccine.

	T0	T2	T3	T4	T5	p value
B	37 (17-126)	43 (15-143)	41 (16-97)	41 (12-121)	47 (14-120)	n.s.
NK	121 (64-253)	140 (84-287)	162 (75-209)	130 (51-222)	160 (69-274)	n.s.
T	751 (436-1230)	996 (616-1421)	1000 (575-1311)	934 (508-1437)	1014 (565-1515)	n.s.
T helper	379 (244-538)	483 (296-710)	497 (256-740)	419 (235-740)	425 (285-771)	n.s.
T cytotoxic	319 (158-559)	409 (220-592)	340 (213-482)	349 (177-611)	397 (203-604)	n.s.

n.s., not significant.

Median and interquartile range.

**Supplemental Figure 1** shows the numbers of memory (**Figure S1A**) and naïve (**Figure S1B**) lymphocytes in patients with TET at T0 compared to those obtained at T2, T3, T4, and T5. The trends of the two lymphocyte populations were complementary, although no significant differences were observed. In fact, memory lymphocyte levels were lower at T4 than at T0, and thus higher at T5 than at T4. Naïve lymphocytes increased at T2, T3, and T4 compared to at T0; thus, they were lower at T5 than at T4.

**Figure 3:**
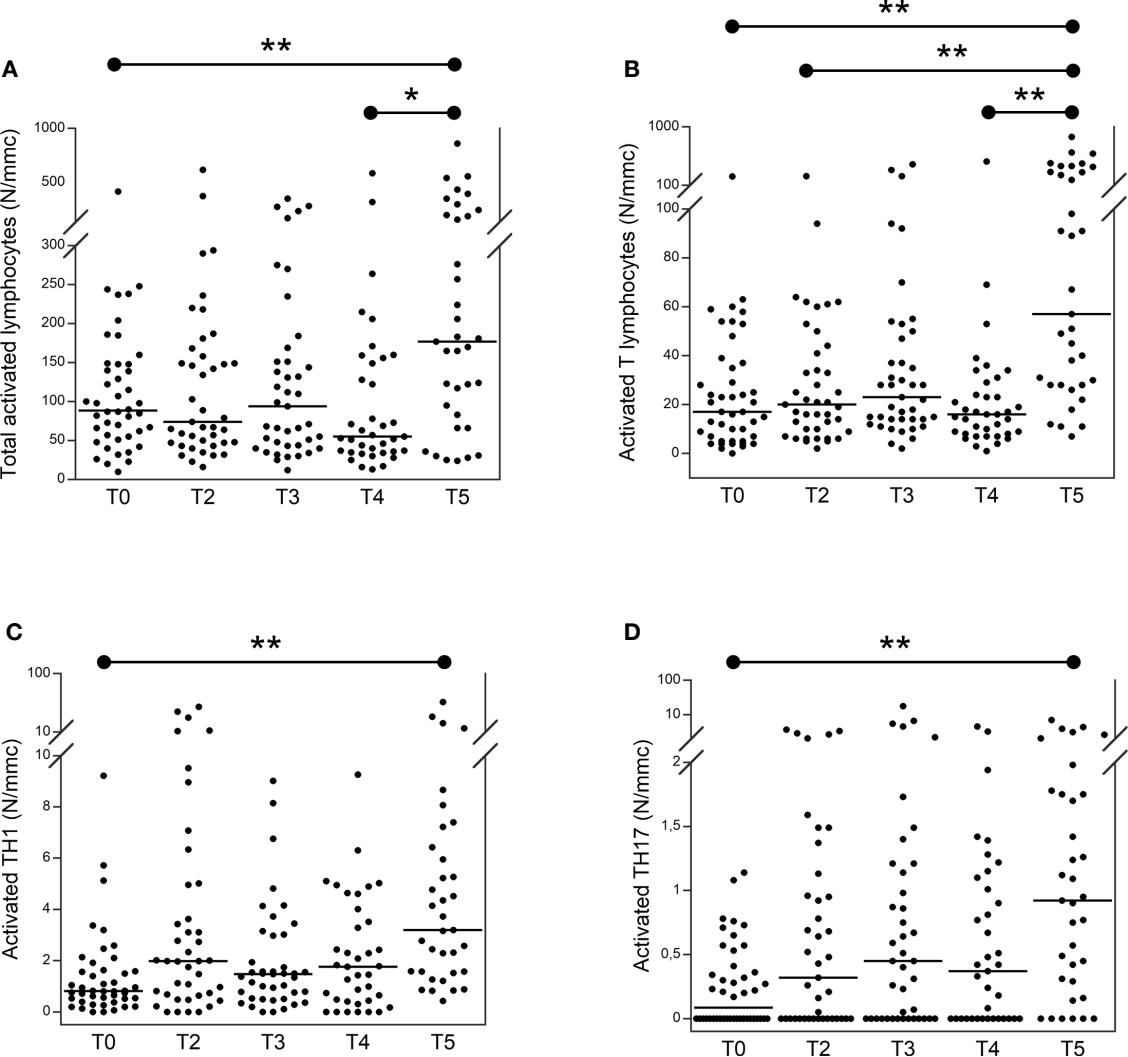
Number of total activated **(A)** activated T **(B)** activated TH1 **(C)** and activated TH17 **(D)** lymphocytes in patients with thymic epithelial tumors at T0 in comparison to T2, T3, T4 and T5. From the lymphocytes, activated T lymphocytes have been identified as CD3+, DR+. From TH cells (CD3+, CD4+), TH17 and TH1 have been identified as CCR6+, CXCR3− and CCR6−, CXCR3+, respectively. CD38+ and HLA-DR+ were used to identify their activated form (23–25). *p<0.01; **p<0.001; N/mmc, number of cells per cubic millimeter.

**Figure 3** shows the trend of activated lymphocytes in patients with TET at T0 compared to those at T2, T3, T4, and T5. The total number of activated lymphocytes (**Figure 3A**) was significantly higher at T5 than at T0 and T4. The number of activated T lymphocytes was significantly higher at T5 than at T4, T2, and T0. The number of activated TH1 (**Figure 3C**) and TH17 (**Figure 3D**) lymphocytes was significantly higher at T5 than at T0.

### Study of correlations between humoral/cellular response and TET clinical/biochemical parameters

**Figure 4:**
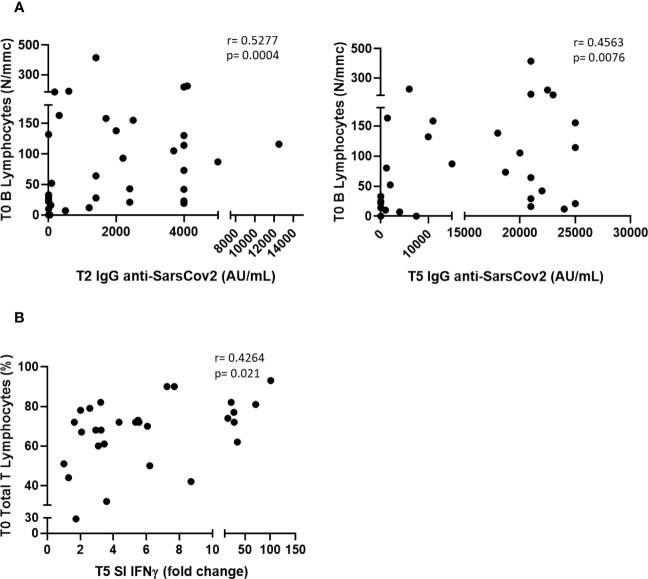
Correlations between serum antibodies level at T2 and T5 and B lymphocytes at T0 **(A)**, and between SI IFN γ at T5 and T lymphocytes (expressed as % of WBC) at T0 **(B)**.

We found a significant difference in IgG anti-RDB levels at T5 between patients with TET with NED and those with ED (21, 000 vs. 1,200 AU/mL, p<0.001; data not shown), while no correlations were observed between the humoral response and other clinical parameters, such as histological type of tumor (thymoma vs. thymic carcinoma), GS, stage of the disease, or autoimmunity. Furthermore, we found significant correlations between the number of B lymphocytes at baseline (T0) and IgG levels at T2 and T5 (**Figure 4A**). We did not identify any correlation between clinical parameters, such as histological type of tumor (thymoma versus thymic carcinoma), NED or ED, GS, stage of the disease or autoimmunity, and IFN γ response. However, we found that the SI of IFN γ at T5 was significantly correlated with the percentage of total T lymphocytes at T0 (**Figure 4B**).

### Multivariate analyses to identify biomarkers discriminating humoral/cellular non-responders

**Figure 5:**
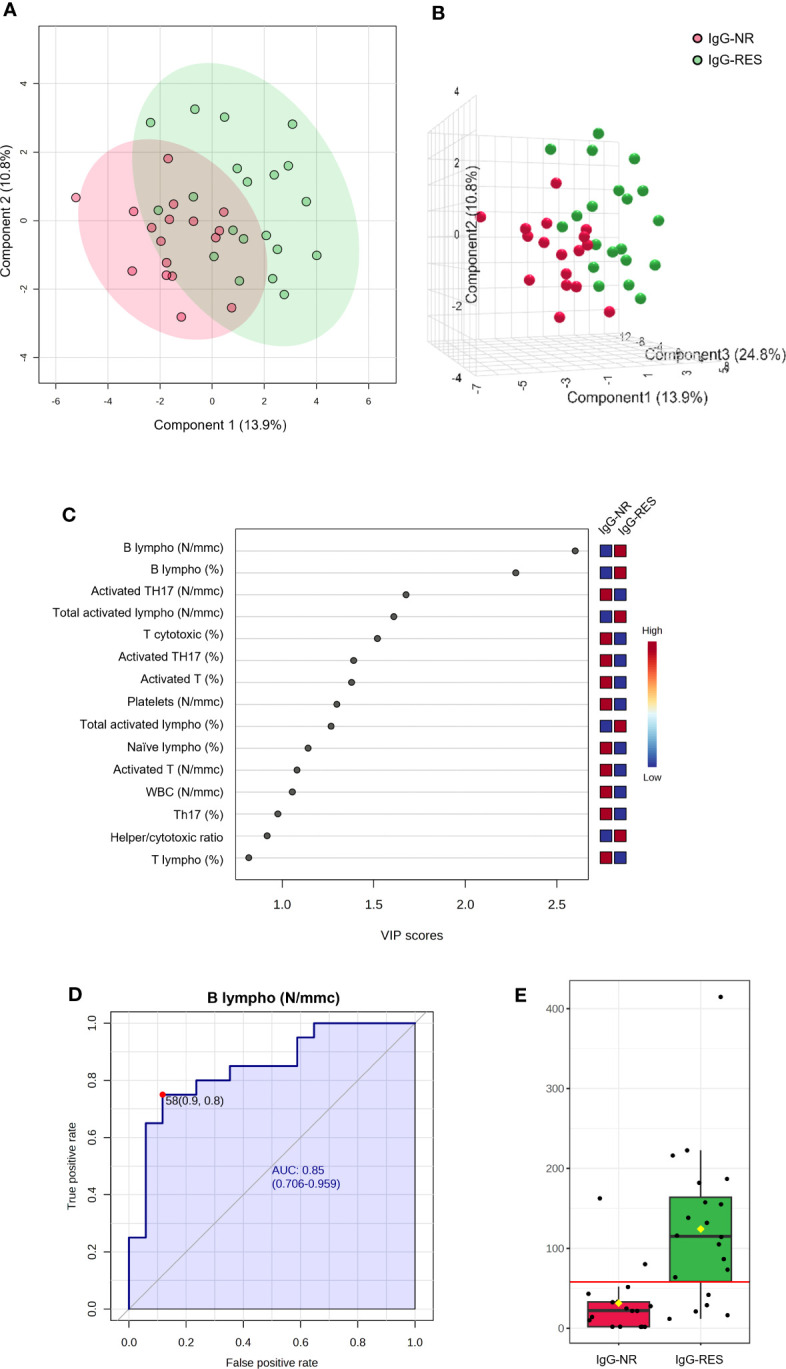
PLS-DA analysis and univariate ROC curve analysis discriminating non responder patients with thymic epithelial tumors for IgG production (IgG-NR) from responders (IgG-RES). **(A)** 2D score plot; **(B)** 3D score plot; **(C)** VIP score of the first 15 features; **(D)** ROC curve for the number of B lymphocytes (B lympho, N/mmc); **(E)** box plot of B lympho (N/mmc) values in IgG-NR and IgG-RES groups (red line represents the best cut-off value).

Among the 44 patients with TET, 17 (38.6. %) did not respond to the production of IgG targeting the RBD (IgG-NR), i.e., the levels of IgG at T5 were undetectable. We performed PLS-DA using the number and percentage values of all white blood cell subpopulations at baseline (T0) in IgG-NR and responder patients (IgG-RES). The PLS-DA 2D score plot showed that the IgG-NR patients clustered in a zone that partially overlapped with the IgG-RES zone (**Figure 5A**). The first (PC1) and second (PC2) components explain 24.7% of the model variance. The PCA-synchronized 3D plot (**Figure 5B**) showed that PC1 and PC2, together with the third component (PC3), separated IgG-NR patients from those with IgG-RES. **Figure 5C** shows the VIP scores of the first 15 variables in the PLS-DA model. Among the variables with a VIP score higher than 2.0, we first determined the number (B lympho, N/mmc) and then the percentage (B lympho, %) of B lymphocytes at baseline, which were both significantly lower in IgG-NR patients than in IgG-RES patients. We then performed a classical univariate ROC curve analysis of B lymphocyte N/mmc. The area under the ROC curve (AUC) was equal to 0.85 (**Figure 5D**) and, according to the criteria of Jones and Athanasiou (26), the B lympho (N/mmc) model shows a “good” AUC. At the best cutoff of 58 N/mmc (**Figure 5E**), the model showed a sensitivity and specificity of 77% and 88%, respectively, in discriminating IgG-NR from IgG-RES patients, and therefore, in the prediction of IgG response to the vaccine.

**Figure 6:**
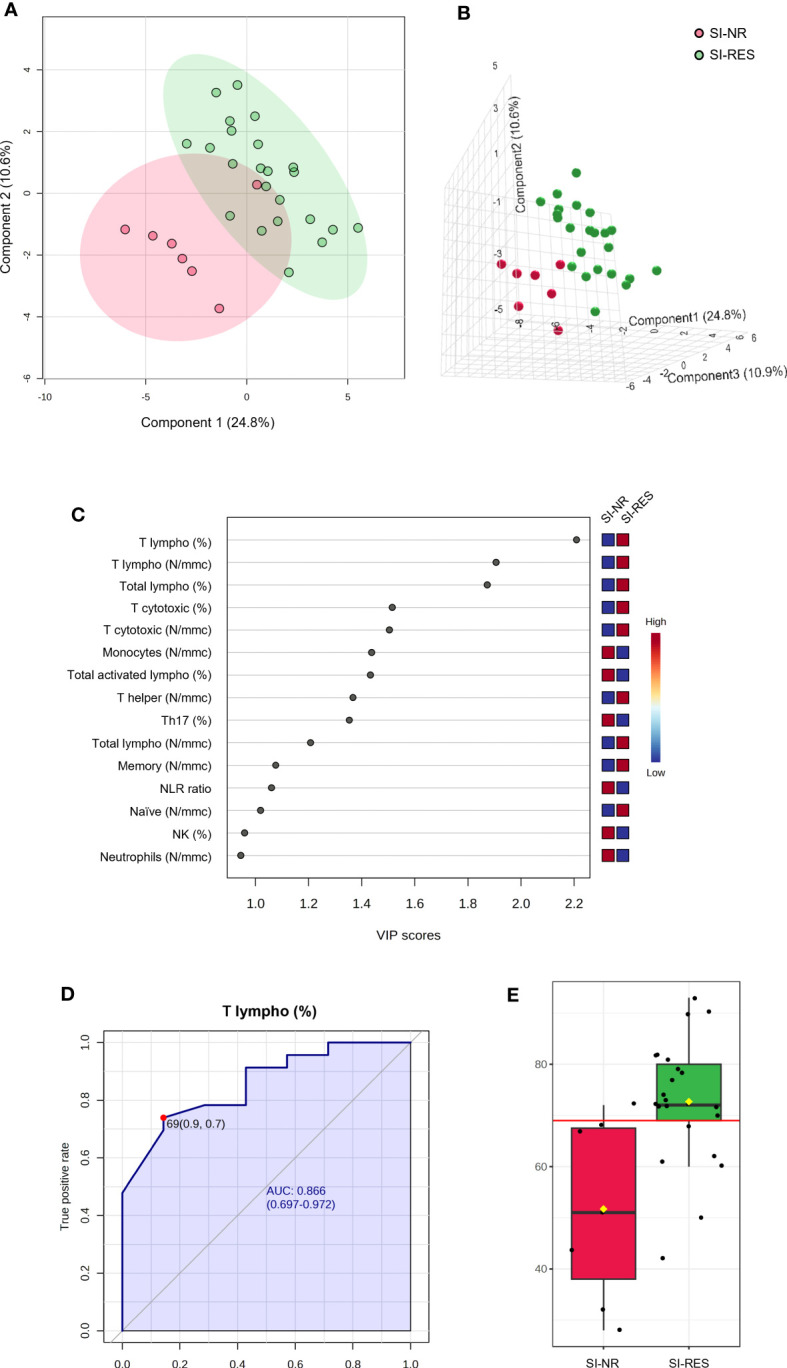
PLS-DA analysis and univariate ROC curve analysis discriminating non responder patients with thymic epithelial tumors for IFN γ SI (SI-NR) from responders (SI-RES). **(A)** 2D score plot; **(B)** 3D score plot; **(C)** VIP score of the first 15 features; **(D)** ROC curve for the percentage of T lymphocytes (T lympho, %); **(E)** box plot of T lympho (%) values in SI-NR and SI-RES groups (red line represents the best cut-off value).

Similarly, seven (15.9%) did not respond to IFN γ SI (SI-NR) among patients with TET. We performed PLS-DA analysis using the number and percentage values of all white blood cell subpopulations at baseline in SI-NR and SI-RES patients (SI-RES). The PLS-DA 2D score plot showed that only 1 patient falls in the SI-RES zone (**Figure 6A**). PC1 and PC2 explain 35.4% of the model variance. In the PCA-synchronized 3D plot (**Figure 6B**), SI-NR patients were completely separated from SI-RES patients. The only variable with a VIP score higher than 2.0 was the percentage of T lymphocytes (T lymphocytes, %, **Figure 6C**). Univariate ROC curve analysis of T lymphocyte (%) showed a “good” AUC of 0.866 (**Figure 6D**). At the best cutoff of 69% (**Figure 6E**), the model showed a sensitivity and specificity of 76% and 86%, respectively, in differentiating SI-NR from SI-RES patients, and therefore, in the prediction of SI response.

### Safety of SARS-Cov-2 vaccine, incidence and outcome of COVID-19 infection occurring during the observation period

Overall, the vaccine was well tolerated and no serious adverse events were observed. At the end of the full vaccine cycle, patient clinical characteristics and the percentage of cases with immune-related paraneoplastic disorders were unchanged (as was their clinical picture).

Nineteen of the 44 enrolled patients (43.2%) developed acute COVID-19 during the observation period. Of these, three patients had COVID-19 between the second and booster doses, and 16 had COVID-19 within 6 months after the booster dose. Only one patient experienced COVID-19 twice during the observation period. Importantly, none of the 19 patients required hospitalization or oxygen support, and no fatalities were observed. Antiviral therapy was prescribed to these patients in April 2022 according to the National Guidelines (27).

Among the 19 patients who experienced COVID-19 during the vaccine cycle, 5 (26.3%) were not responders at the humoral level (i.e., they did not develop IgG), while only one patient did not respond at the cellular level (i.e., he did not develop an IFNγ response). The chi-square test excluded any significant differences between the responders and non-responders in the risk of being affected by COVID-19.

## Discussion

In our study population of 44 patients with TET, the COVID-19 vaccine was safe, with no cases of worsening autoimmunity or other severe complications. The vaccine protects against the development of severe SARS-Cov-2 infection. Most patients exhibited humoral (61.4%) and/or cellular (84.1%) responses, particularly after the third booster dose. The numbers of B and T lymphocytes at T0 predicted effective humoral and cellular immunization, respectively. After the booster dose, we observed a significant increase in the number of activated lymphocytes, particularly TH1 and TH17.

A previous report (28) described the absence of autoimmune reactivation in 126 patients with TET, 30% of whom had autoimmune diseases and received at least one dose of the SARS-CoV-2 vaccine. Another study reported autoimmune flares in three patients with TET after the first dose and in three after the second dose; however, in all cases, such events were mild and self-limiting, thus concluding on the safety of the COVID vaccine in patients with TET (29). Our data suggests that a lack of autoimmune reactivation in 44 patients with TET, 77% of whom had preexisting autoimmune diseases. Furthermore, the follow-up of our patients excluded any worsening of MG due to the vaccine in the 13 patients previously affected by such complications or the appearance of MG in the 31 patients who were free from such complications before the cycle. These data were compared with 2/126 cases that developed MG after vaccination (28) and 2/22 cases of MG in which such complications worsened after vaccination (30). Six patients with TET developed COVID-19 after the second dose of the vaccine and eight within 6 months following the booster dose. None of the 14 patients required hospitalization or oxygen supplementation. This confirms that in patients with TET, the vaccine does not prevent the infection, likely due to the different VOI of the virus that spread in Italy (20). However, it avoided the severe form of the disease, as was observed in healthy subjects (20, 31). Moreover, as observed in healthy subjects (20), humoral and cellular responses to the vaccine are not predictive of a reduced risk of infection.

The most significant result of our study was the extraordinary enhancement of the immune response induced by the booster dose. In fact, the level of serum IgG anti-SARS-CoV-2 had already significantly increased after the second dose of the vaccine and declined within 6 months. After the booster dose, this level was enhanced with a response that appeared increasingly similar to that observed in normal subjects after the booster dose (20). Our data are consistent with a study on 47 multiple sclerosis patients treated with ocrelizumab or fingolimod (32). In these patients a weaker and shorter humoral response to the first two doses of BNT162b2 mRNA SARS-CoV-2 vaccine was observed, while the booster vaccine dose was able to enhance the level of serum IgG anti-SARS-CoV-2 together with a good safety and tolerability profile. In addition, Wroński et al. (33) observed an increase in the humoral response after the booster dose in 49 patients with inflammatory arthritis, although the antibody levels were lower compared to healthy subjects. This study also demonstrated an increase of IFNγ release after the booster dose, although this release was significantly lower than that observed in healthy subjects. The release of IFNγ after cell stimulation (SI index) is the most effective biomarker of the cell response, as observed in healthy subjects (20), confirming the role of IFNγ against SARS-CoV-2 (34–36). While, TNF α SI and IL-2 SI seem to be less effective as biomarkers of cell immunization, in disagreement with previous studies that reported a significant role of TNFα and of T cells expressing IL-2 toward the virus (37–39). No other studies are available on the humoral and cellular responses to the COVID-19 vaccine in patients with TET. However, our data are consistent with the results of a study of 46 patients with metastatic solid malignancies undergoing active treatment in which humoral response was highly variable and unstable after two vaccine doses, whereas the booster dose led to a significant enhancement of both humoral and cellular responses, not influenced by baseline factors and treatment type (40). On the other hand, a previous study of 20 patients with immunodeficiency (mostly humoral) demonstrated that the booster dose enhanced the cell response but did not influence the humoral response (41). Moreover, a study on people living with HIV showed that humoral response after the booster dose was strong and higher than that achieved with the second dose, but the cellular response remained stable (42). In any case, we confirm that the IFN γ response and the levels of serum antibodies are not correlated.

Among the 44 patients with TET included in the study, 17 did not develop any humoral response and seven did not develop any IFNγ response after the booster dose. Only the evidence of the disease at T0 was related to the humoral response (i.e., patients with ED developed a significantly lower antibody level after the booster dose compared to those with NED), while all the other clinical parameters (i.e., type and stage of the disease, AD, and GS) were not related to the humoral response after the booster dose, and no clinical parameters were related to the IFN γ response after the booster dose. These findings confirm our previous study reporting a significant association between the impaired seroconversion at 1 month after second dose and ED in TET patients (43). Interestingly, our multivariate analysis indicated that B and T lymphocytes at T0 were highly effective in predicting humoral and cellular responses, respectively, after the booster dose. Our data agree with a report on 20 patients with multiple sclerosis under fingolimod treatment in which the number of B lymphocytes at T0 was predictive of the humoral response after three doses of the COVID vaccine (44); however, no other studies are available to compare our data. Several studies, including a systematic review (45), have highlighted that a fourth booster dose improves the antibody response against COVID-19 either in patients who already responded to the third dose or in patients who had a weak humoral response. We do not agree with this simplistic view because the antibody title is not predictive of effective immunization against SARS-CoV-2 infection. Our study and other studies strongly support the monitoring of both humoral and cellular responses to the COVID-19 vaccine in immunocompromised subjects (46). Furthermore, we suggest a preliminary analysis of B and T cells in patients with immunodeficiency to predict candidates for effective immunization after the vaccine and those that may benefit from alternative preventive approaches (47). This procedure may be extended to other vaccination protocols for patients with frailty.

Interestingly, the booster dose caused a significant increase in the number of activated lymphocytes (i.e., total activated, T-activated, activated TH1, and TH17 lymphocytes) in patients with TET. This induction was specific, as all other lymphocyte populations (i.e., B and T, NK, memory, naïve, helper, and cytotoxic, and total TH1 and TH17) were not modified, as it did not modify the number of WBC, total lymphocytes, neutrophils, and platelets. A study of 26 patients with Sjogren’s syndrome reported that the COVID-19 vaccine did not change the B and T lymphocyte populations; however, activated lymphocytes were not evaluated (48). The evaluation of 335 healthy volunteers (49) demonstrated an increase in activated TH2 lymphocytes and poor activation of TH1 after two doses of the vaccine, but no data are available after the booster dose (which in our patients caused the most relevant increase in activated TH1 and TH17), while a relevant TH1 response to the vaccine was reported either in blood (50) or nasal cells (1). No studies on activated lymphocyte populations in frail patients following COVID-19 vaccination are available. Thus, it is difficult to conclude whether the increase observed in patients with TET is a favorable element of the response to the vaccine or if it may trigger an inflammatory response, considering the plethora of cytokines and other proinflammatory molecules released by TH1 and TH17 activated cells.

The main limitation of our study is the small sample size, although it should be considered that TETs are extremely rare diseases and that study subjects were clinically homogeneous and comprehensively evaluated during the whole vaccine cycle with clinical and flow cytometry assessments.

To the best of our knowledge, this is the first report to demonstrate the effect of the SARS-Cov-2 mRNA vaccine on both humoral and cellular responses in patients with TET.

In conclusion, our data strongly support the use of the SARS-Cov-2 vaccine in patients with TET as it is safe and prevents severe COVID-19 (51). Moreover, we found that the booster dose was particularly effective in determining a humoral immune response. However, a moderate percentage of patients with active tumor lesions did not show a seroconversion even after the full vaccine cycle, and baseline analysis of B and T lymphocytes may help to identify those patients who have lower probability to achieve effective humoral and cellular responses. These findings suggest the importance of monitoring both humoral and cellular responses (through the analysis of IFN γ SI) and analyzing baseline levels of B and T cells to select patients who have a higher probability of receiving the vaccine and select those who may benefit from passive immunization.

We believe that our findings have important clinical implications for defining effective immunization strategies, particularly in frail patients such as those suffering from TETs.

## Data availability statement

The raw data supporting the conclusions of this article will be made available by the authors, without undue reservation.

## Ethics statement

The studies involving humans were approved by Ethical Committee of the University of Naples Federico II. The studies were conducted in accordance with the local legislation and institutional requirements. The participants provided their written informed consent to participate in this study.

## Author contributions

GCr and MGz performed the experiments, analyzed the data, and drafted the manuscript. GS carried out the experiments and analyzed the data. EP, MT, PDP, and MO contributed to patient enrolment, clinical data collection, and data analysis. MR performed the experiments. The GP contributed to patient enrollment, data analysis, and interpretation of the results. PF contributed to data analysis and interpretation of results. GC and MG planned and supervised the study, performed data analysis and interpretation of the results, and drafted the manuscript. All authors contributed to the article and approved the submitted version.
