# Supplementary figures and images for: Immunocytometric analysis of patients with thymic epithelial tumors revealed that COVID-19 vaccine booster strongly enhanced the immune response
Source: Front Immunol. 2023 Aug 29;14:1233056. doi: 10.3389/fimmu.2023.1233056 (PMC10495582; doi:10.3389/fimmu.2023.1233056)

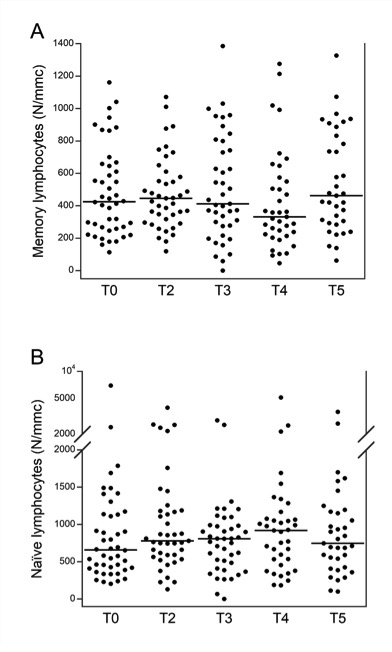

Supplement: Supplementary file 1 [file Image_1.jpeg]
