# Supplementary material for: Immunocytometric analysis of patients with thymic epithelial tumors revealed that COVID-19 vaccine booster strongly enhanced the immune response
Source: Front Immunol. 2023 Aug 29;14:1233056. doi: 10.3389/fimmu.2023.1233056 (PMC10495582; doi:10.3389/fimmu.2023.1233056)
Supplement: Supplementary file 2 [file Table_1.docx]

Supplemental Table 1. White Blood Cells (WBC) and platelets (N/mmc) in patients with TETs from baseline to post-third dose of COVID-19 vaccine. Median and interquartile range.

_______________________________________________________________________________________________________________________

**T0 T2 T3 T4 T5 p value**

_______________________________________________________________________________________________________________________

WBC 6970 (5257-7632) 6860 (5510-8850) 6440 (5000-7795) 6630 (5175-8970) 6790 (5370-9260) n.s.

Neutrophils 4564 (3437-5829) 4617 (3564-6788) 4310 (3009-5871) 4391 (3092-6556) 4521 (3650-6596) n.s.

Lymphocytes 1121 (696-1727) 1305 (924-1812) 1251 (897-1592) 1266 (728-1852) 1371 (729-1887) n.s.

Platelets 253 (184-281) 255 (187-295) 261 (193-298) 248 (185-332) 228 (176-312) n.s.

_______________________________________________________________________________________________________________________

n.s.: not significant.
